# Expression Analysis of a Stress-Related Phosphoinositide-Specific Phospholipase C Gene in Wheat (*Triticum aestivum L.*)

**DOI:** 10.1371/journal.pone.0105061

**Published:** 2014-08-14

**Authors:** Ke Zhang, Congcong Jin, Lizhu Wu, Mingyu Hou, Shijuan Dou, Yanyun Pan

**Affiliations:** College of Life Science, Agricultural University of Hebei, Baoding, China; Institute of Genetics and Developmental Biology, Chinese Academy of Sciences, China

## Abstract

Plant phosphoinositide-specific phospholipases C (PI-PLCs) function in several essential plant processes associated with either development or environmental stress. In this report, we examined the expression patterns of *TaPLC1* under drought and high salinity stress at the transcriptional and post-transcriptional levels. *TaPLC1* mRNA was expressed in all wheat organs examined. U73122 and edelfosine, the PLC inhibitor, impaired seedling growth and enhanced seedling sensitivity to drought and high salinity stress. Though *TaPLC1* expression in wheat was lowest at the seedling stage, it was strongly induced under conditions of stress. When 6-day-old wheat seedlings were treated with 200 mM NaCl or 20% (w/v) PEG 6000 for 6 or 12 h, respectively, the *TaPLC1* transcript level increased by 16-fold compared to the control. Western blotting showed that the TaPLC protein concentration was also maintained at a high level from 24 to 48 h during stress treatment. Together, our results indicate the possible biological functions of *TaPLC1* in regulating seedling growth and the response to drought and salinity stress.

## Introduction

The ability to respond to a variety of abiotic stress signals is crucial for plants. Studying the functions of stress-related genes is critical in order to understand the molecular mechanisms of stress tolerance in plants [1], [2]. In response to high salinity and drought stress, the expression of various genes involved either directly or indirectly in plant protection is altered. The products encoded by these genes include osmolytes, ion channels, receptors, calcium signaling components, and other regulatory signaling factors or enzymes [3]. Several studies have demonstrated the important role of the phosphoinositide signaling pathway at multiple developmental stages and in response to environmental stress in plants [4]–[6]. Phosphoinositide-specific phospholipases C (PI-PLCs, PLCs) are essential enzymes in phosphoinositide signaling. PLC hydrolyzes phosphatidylinositol 4,5-bisphosphate (PIP2) upon activation, generating inositol 1,4,5-trisphosphate (IP3) and 1,2-diacylglycerol (DAG), both of which are second messengers in the phosphoinositide signal transduction pathway [7]. PI-PLC can act on phosphatidylinositol 4-bisphosphate (PI4P) *in* *vitro*. Plant PI-PLC signalling is differences and similarities to the mammalian paradigm. Higher plants lack IP3 receptor, a ligand-gated Ca^2+^ channel, and PKC. Instead, plants seem to use their phosphorylated products, IP6 and PA, as signaling molecules [4], [6]. The first plant PLC gene to be identified was cloned from *Arabidopsis thaliana* shoots, and it was found to exhibit a high degree of sequence similarity to animal *PI-PLC* genes [8]. Subsequently, Hirayama et al. [9] obtained a cDNA (*AtPLC1*) from *A. thaliana* shoots exposed simultaneously to dehydration and salt stress. To date, plant PLCs have been cloned in many plant species, including oat [10], [11], soybean [12], potato [13], *Nicotiana rustica* [14], mung bean [15], *Lilium* [16], *Petunia* [17], rice [18], maize [19], and tomato [20], [21]. The role and regulation of PLC isozymes have been well established in animals [22], and their mode of action is considered to be different from that of plant PLCs. Despite conclusive data concerning the roles of PLCs in plant cells, little is known about their *in planta* regulation [23].

Wheat (*Triticum aestivum*) is one of the most important staple foods in the world. Drought and salt affect wheat plant growth and productivity, and reduce yields worldwide [24]. Evidence indicates that wheat PLCs (TaPLCs) play a role in the response of plants to abiotic stimuli. As early as 1992, PI-PLC activity was detected in the plasma membrane of wheat root cells [25], [26]. Wheat PI-PLC is similar to that in other plants in that the substrate of TaPLC hydrolysis is phosphatidylinositol 4-phosphate (PIP) and PIP2, and TaPLC is dependent on calcium ions for activity [25]. Jones and Kochian [27] showed that the phytotoxic metal cation Al^3+^ inhibited root growth. AlC1_3_ and AI-citrate specifically inhibited PLC action in a dose-dependent manner and at physiologically relevant AI levels. Cerebroside C increases tolerance to chilling injury and alters the lipid composition of wheat roots, possibly due to a partial reduction in lipid peroxidation and alterations in lipid composition, including inhibition of the activities of PLC and phospholipase D (PLD) [28]. There are two *PLC* genes in the wheat genome: *TaPLC1* and *TaPLC2* (GenBank: HM754654.1 and HM754653.1). *TaPLC1* was recently shown to interact with Gα and to be involved in the response to cold stress in wheat [29]. In this study, we analyzed the expression patterns of *TaPLC1* in wheat plants exposed to salt and drought stress in order to provide data for the rational engineering of hardier versions of this plant.

## Materials and Methods

### Plant culture and treatments

Seeds of wheat (Chinese Spring background) were briefly surface-sterilized in a solution of 70% (v/v) ethanol, followed by immersion in a 30% (v/v) commercial bleach solution for 10 min. They were then washed with sterilized water three times. Wheat plants were grown and maintained using a hydroponic system. Plates were incubated in a growth chamber under 16 h of light at 22°C.

For high salinity treatment and drought treatment, respectively, NaCl or PEG 6000 was added to the nutrient solution at increasing concentrations up to 200 mM NaCl or 20% PEG 6000. Wheat seedlings treated with various chemicals and stress elicitors along with control plants were sampled at 0.5, 1, 2, 6, 12, 24 and 48 h post-treatment. In addition, various tissues, including roots, stems, leaves, and ear, were sampled at different developmental stages. All samples were rapidly frozen in liquid nitrogen and stored at −80°C.

The shoot length, fresh weight of stressed-seedlings and several relevant physiological parameters were measured. Relative water content (RWC) was measured by the Saturated weighing method [30]. The content of chlorophyll (CHL) was determined by Hegedüs et al. [31]. Malondialdehyde (MDA) content was measured by the method of Dhindsa et al. [32]. The data were statistically analyzed by one-way Analysis of Variance (ANOVA).

### PLC inhibitors treatment and growth measurement

PLC inhibitor, U73122 and its inactive form, U73343, were purchased from Sigma-Aldrich (Madison, WI). They were freshly prepared in DMSO. Another PLC inhibitor, edelfosine, were from EMD Chemicals, Inc. and was freshly prepared in water.

Two kinds of PLC inhibitor were used to determine the role of *TaPLC1* during germination and at the seedling stage, respectively. Wheat seeds were treated with medium containing 15 µM U73122 or 100 µM edelfosine, and 15 µM U73343 or water along with control seeds, and germination rate recorded in the next three days. Germination seeds were treated with same condition and subsequently transferred to medium for testing the seedlings growth. To determine the role of *TaPLC1* under salt stress and drought stress, U73122 and U73343, edelfosine and water, were injected into the leaves of seedlings as described previously, respectively [33]. Three biological replicates were conducted.

### RNA extraction and gene expression analysis

Two-week-old wheat seedlings (for seedling, root, and leaf tissues) were collected for *TaPLC1* expression analysis. Total RNA was isolated using TRIzol reagent (Life Technologies, Carlsbad, CA). Contaminating DNA was eliminated with DNase I (TaKaRa) treatment, and reverse transcription was performed using M-MLV reverse transcriptase (TaKaRa). Real-time RT-PCR was conducted in triplicate on the Bio-Rad Chromo4 real-time PCR system using the SYBR Green PCR master mix (TansGen Biotech). Real-time RT-PCR was conducted to determine the *TaPLC1* transcript level using the primers *TaPLC1*RT-F (5′-CGTGCTCCTATCAACAAAGCC-3′) and *TaPLC1*RT-R (5′-CTGTTCGTCCTCATCGTCGT-3′). 18S rRNA was used as a quantitative control and amplified using the primers T-18S-F (5′-GCATTTGCCAAGGATGTTTTC-3′) and T-18S-R (5′-TGCTATGTCTGGACCTGGTAAGT-3′). The line arrangement of the products was established by comparing samples that were run for different numbers of cycles. Three biological replicates were conducted and the results were analyzed with SPSS statistics 17.0 (IBM) using the independent-samplest test. Real-time RT-PCR analysis of the transcript levels of *TaPLC1* in different time after treatment was compared to 0 h which was set to 1.0.

### Protein extraction and Western blotting

Proteins were extracted then separated by SDS-PAGE and electrophoretically transferred to a PVDF membrane as described [34]. Immunodetection was performed as described by Parre et al. [35]. Antisera were obtained by the immunization of rabbits with TaPLC1 (amino acids 151–350). The blots were washed with PBS-T. Detection was performed by an ECL assay using horseradish peroxidase-conjugated antibodies. Equal protein loading was verified using anti-actin antibodies (provided by Dr. Guozhen Liu, Hebei Agricultural University, Baoding, China).

### Preparation of anti-TaPLC1 antibodies

To prepare anti-TaPLC1 antibodies, a 600-bp fragment (Text S1A) which is conserved in *TaPLC1*, was amplified from wheat genomic DNA using the primers Anti-PLC1 F (5′-CGGAATTCGCCAAGGATGGTGGTGCCGC-3′) and Anti-PLC1 R (5′-CCGCTCGAGTTTTGGATCGAAAACTTCCGGC-3′) and translated to TaPLC1 (amino acids 151–350) (Text S1B). The PCR products were cloned into PMD19-T simple vector. Sequencing was conducted to ensure the integrity of the clone. The pMD19-T-*TaPLC1* plasmid, digested with *Eco*RI and *Xho*I, was identified by electrophoresis (Figure S1A).

To express TaPLC1 (amino acids 151–350), the products of pMD19-T-TaPLC1 plasmid, digested with *Eco*RI and *Xho*I, were cloned into pET-30a. To identify the proteins, SDS-PAGE was performed (Figure S1B).

To prepare anti-TaPLC1 antibodies, the TaPLC1 fragment (amino acids 151–350), purified from bacteria (Figure S1C), and was sent to Beijing protein institute (BPI). The antibody titer is shown in Table S1.

It should be noted that we generatean antibody that is not expected to completely discriminate between the different PLC isoforms. According to the software analysis, the TaPLC1 epitope is partially within the TaPLC2, the anti-TaPLC1 can also recognize the TaPLC2 and weakly cross with TaPLC2.

## Results

### Phylogenetic analysis and expression patterns of *TaPLCs*

**Figure 1:**
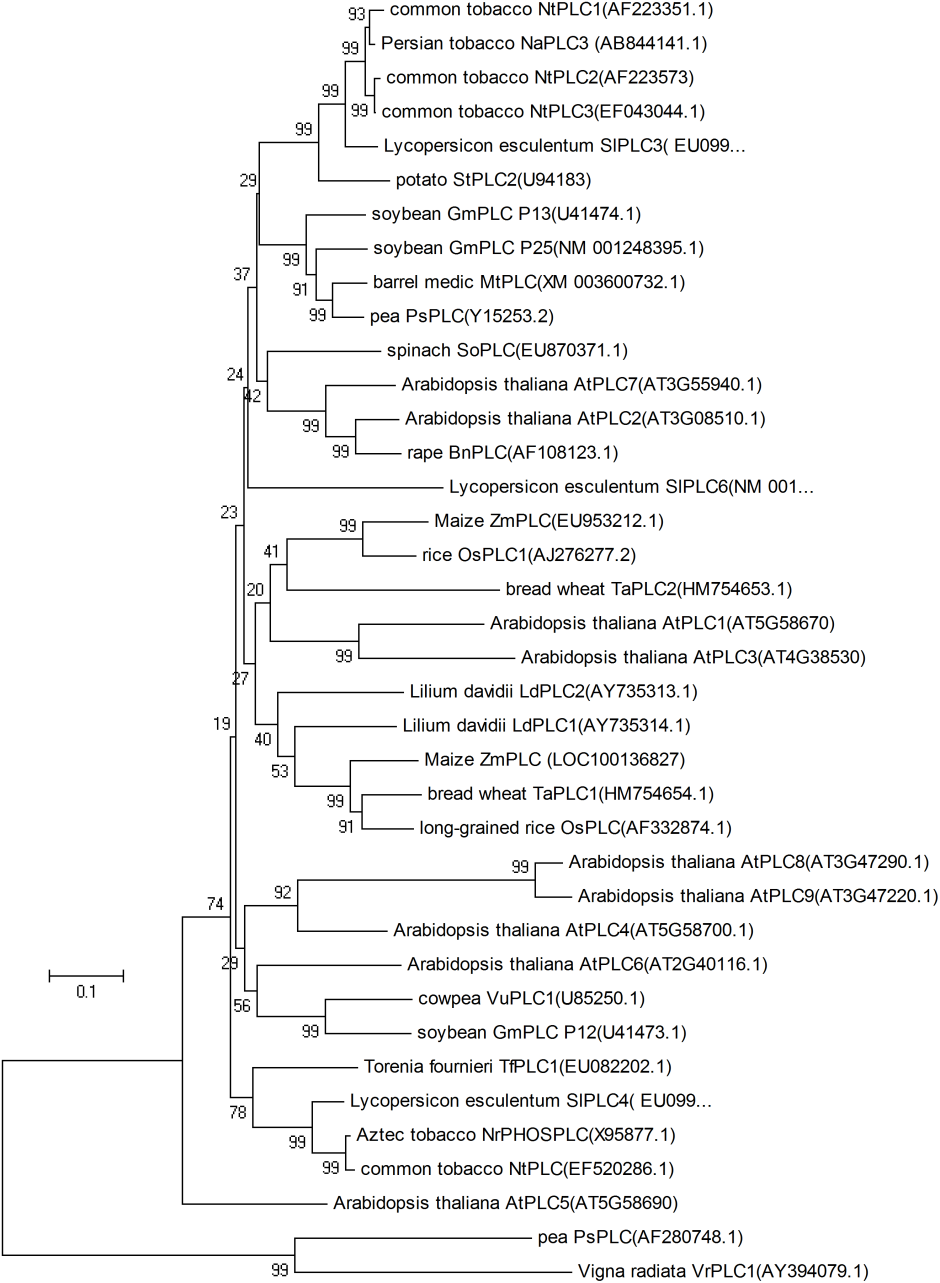
Phylogenetic analysis of *TaPLCs*. Phylogenetic analysis of *TaPLCs* among plant *PI-PLCs*. The sequences of the *PLCs* were obtained from the NCBI and aligned using MEGA. An unrooted phylogenetic tree was created using the maximum likelihood method in PhyMl (WAG substitution model). The numbers at the nodes represent the values of neighbor joining bootstrap support obtained using the approximate likelihood-ratio test. Branches below 50% were collapsed. The scale bar represents the number of substitutions per site. Unclassified proteins were named according to maximum similarity with the respective *PI-PLC* isozyme of *Arabidopsis*.

The *T. aestivum* genome carries two *TaPLC* genes; they possess the relatively simple structure of all plant PI-PLC, including X/Y catalytic domains, a C2 domain, and truncated EF-hand domains (Pokotylo et al., 2014). All higher plant PLCs share a common ancestor and are phylogenetically separated from human and yeast PI-PLC proteins [36]. We conducted a phylogenetic analysis of TaPLCs using plant PLC enzymes and found that the PLCs of monocots are separated from those of dicots (Figure 1). It is interesting that the *A. thaliana*, has the smallest genome of known plants genomes, contains the most members of the PLC family based on current data, and that the two AtPLC isoforms (AtPLC1/3) were grouped together with the PLCs of monocots. Phylogenetic comparison suggests a single origin of the AtPLC and OsPLC genes, with subsequent expansion of the gene family by duplications [37]. Pokotylo et al. [30] think that progenitors of AtPLC1/AtPLC3 possibly arose from a single gene by a duplication event on chromosome 5, with a subsequent duplication and relocation of AtPLC3 to chromosome 4. Our phylogenetic analysis further shows that AtPLC1/3 might represent an evolution closely with monocots.

**Figure 2:**
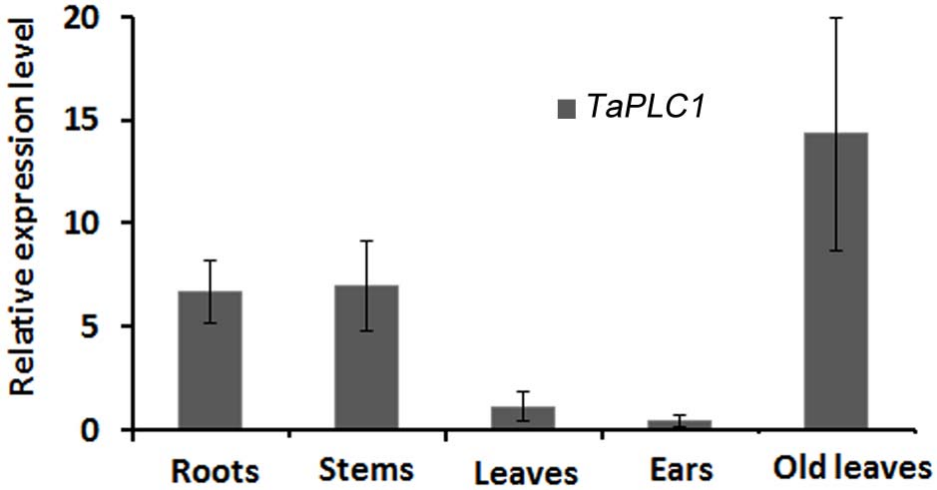
The relative expression patterns of *TaPLC1* in different organs of *Triticum aestivum*. To determine the expression pattern of *TaPLC1*, roots, stems, leaves (grown for 6 days), ears, and old leaves (booting stage) were sampled at different developmental stages. Three biological replicates were conducted.

A real-time RT-PCR approach was applied to investigate the relative expression patterns of *TaPLC1* in various organs of *T. aestivum*. Our data show that *TaPLC1* was expressed in all organs examined, but that the expression levels varied. *TaPLC1* transcripts were expressed at a higher level in root and stem than in seedling and ear. Notably, a large difference in expression level was detected between seedlings and old leaves (Figure 2). This might imply the participation of *TaPLC1* in the aging process.

### The inhibition of PI-PLC decreased seedling growth and resistance to salt and drought stress

**Figure 3:**
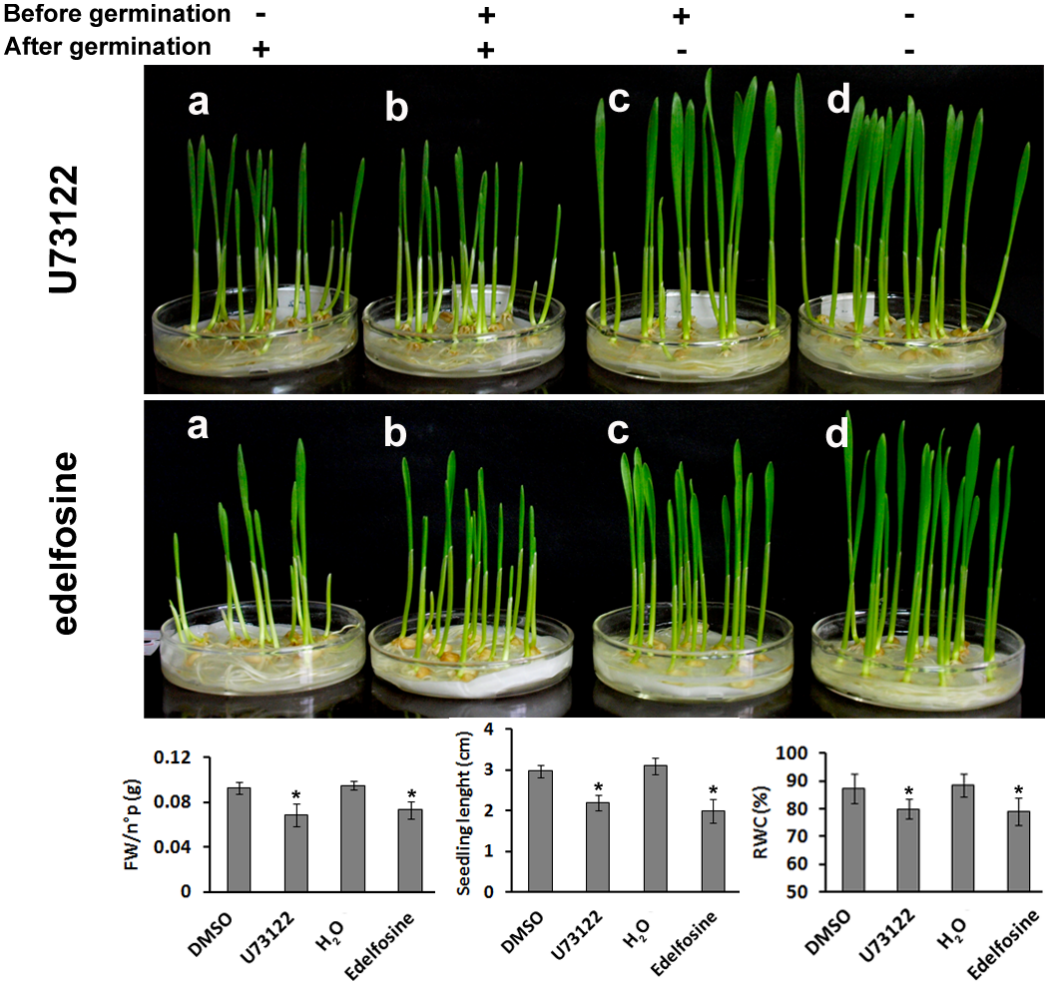
TaPLC is involved in wheat seedling growth. Pharmacological experiments were used to test the *TaPLCs* regulating the growth of wheat. Two PI-PLC inhibitors, U73122 and edelfosine were used to treat the seeds and germinated seeds, respectively and the state of the seedlings after three days of growth were recorded. a, PI-PLC inhibitor was added on germinated seeds (− +); b, Inhibitor was added on un-germinated seeds, and remained in the culture medium (+ +); c, Inhibitor was added on un-germinated seeds, but removed from culture medium before the seeds germination (+ −); d, Control i.e. DMSO solution or water was added on germinated seeds (− −). Three biological replicates were conducted.

**Figure 4:**
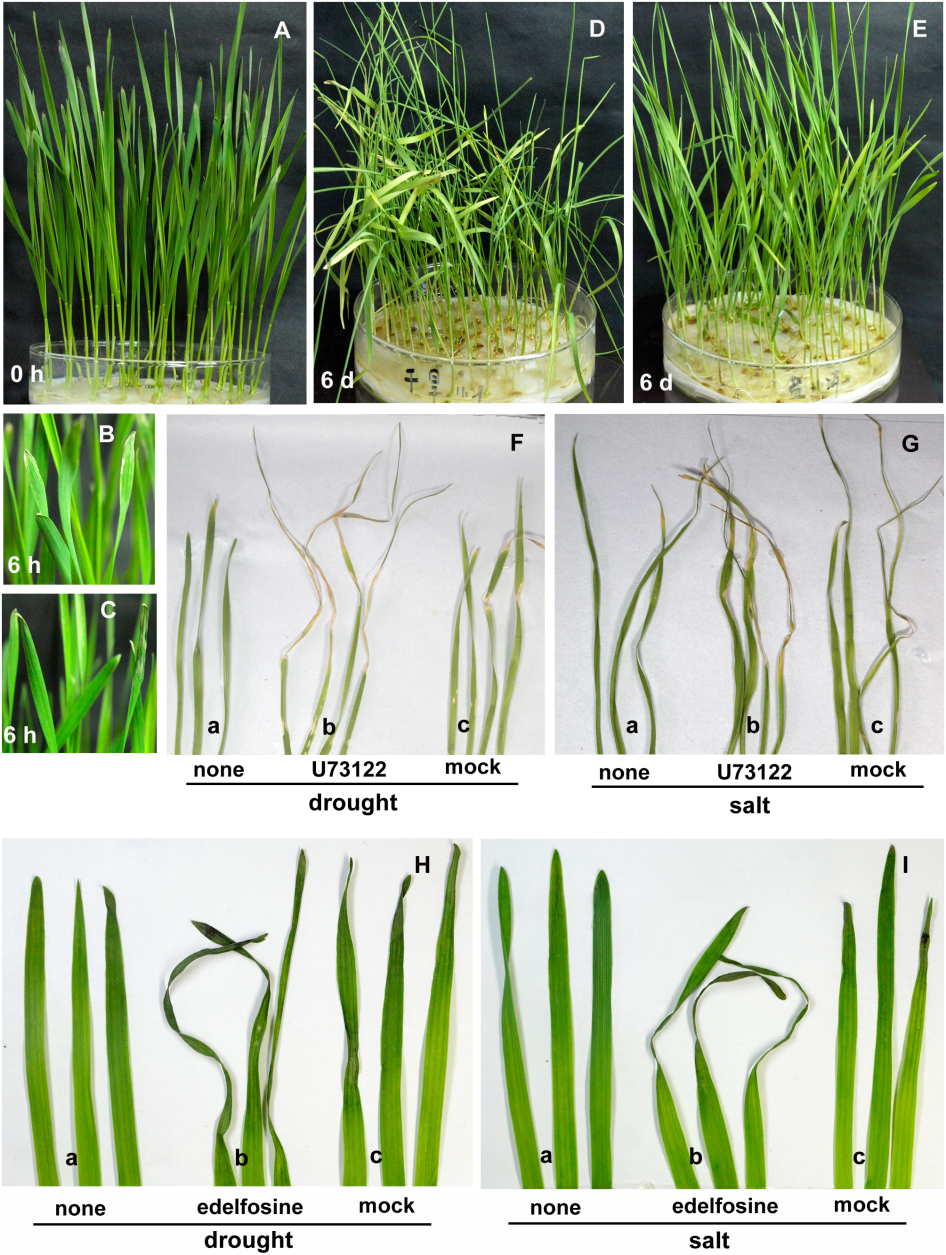
The response of *Triticum aestivum* seedlings to salt and drought stress and treatment with PI-PLC inhibitors. (A) Seedlings 6 days after sowing. (B) The leaf apex of wheat treated with 20% PEG 6000 after 6 h. (C) The leaf apex of wheat treated with 200 mM NaCl after 6 h. (D). Seedlings treated with 20% PEG 6000 after 6 d. (E) Seedlings treated with 200 mM NaCl after 6 d. (F and G) Under drought stress (F) and salt stress (G), those leaves injected with U73122 (b) showed enhanced sensitivity compared to those injected with U73343 (c) or water (a). (H and I) Under drought stress (H) and salt stress (I), those leaves injected with edelfosine (b) showed enhanced sensitivity compared to those injected with water (c) or un-injected (a). Three biological replicates were conducted.

**Figure 5:**
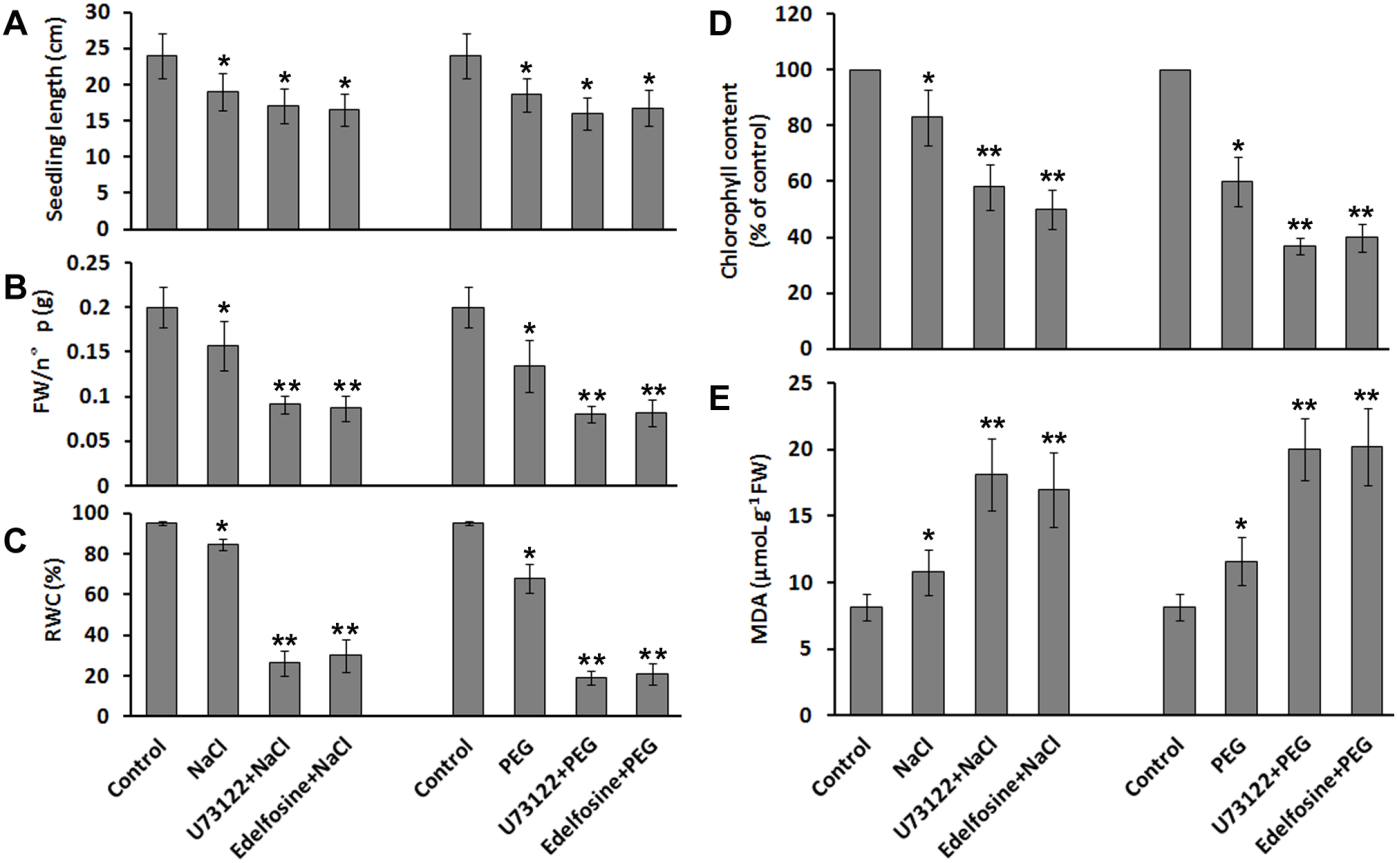
Relevant parameters of *Triticum aestivum* seedlings treated with salt/drought and PI-PLC inhibitors after 6 d. (A) The seedlings lengths. (B) Fresh weight (FW), n°p seedling number. (C) Relative water content (RWC). (D) chlorophyll content of % control. (E) Malondialdehyde (MDA) content. All data were measured three times and statistically analyzed by one-way Analysis of Variance (ANOVA). * indicate significance at *p*<0.01.

Plant PLCs play roles both in regulating development-related processes and responses to stress. In plant development, they have been well characterized during polarized pollen growth. In our study, due to the large difference in expression between seedlings and old leaves, we examined the effects of TaPLC on seedling growth. U73122, a specific inhibitor of PI-PLC [38], is added on seeds or seedlings that the germination has already taken place and the radicle can be seen. After 3 days, the treated seeds and seedlings were both about 2 cm in length and fresh weight of each seedling was 0.068 g (Figure 3), whereas the control seedlings, which were treated with U73343, an inactive analog of U73122, or DMSO, U73122 solvent, were already 3 cm in length and fresh weight of each seedling was 0.092 g (Figure 3). When the treated-seeds with U73122 begun to emerge the radicle, the inhibitor was removed from the medium and the seedlings have also grown to 3 cm after 3 days (Figure 3). These result showed that seedling growth rate was significantly reduced by U73122, whereas U73122 had no effect on seeds germination. Edelfosine, another PI-PLC inhibitor [39] was also be used to testing the seeds germination and seedlings growth. The similar results were obtained (Figure 3). These results indicate that TaPLC is involved in the regulation of seedling growth. The seedlings resumed a normal growth rate when U73122 was removed from medium (data not showed). In addition, the results did not show the inhibition for U73122 or edelfosine on seeds germination. We further tested seeds germination using the two inhibitors and confirmed this result (Figure S2). PI-PLCs are often considered to be stress-activated enzymes [36]. We therefore examined the regulatory function of TaPLCs in wheat seedling growth during salt and drought treatment. Following treatment for 6 h with 200 mM NaCl or 20% PEG 6000, the leaf apex of the wheat seedlings began to wilt (Figure 4B and C). Six days later, the growth of the seedlings was severely impaired, leaves began to curl and yellow (Figure 4D and E). In comparison, the seedlings pre-treated with U73122 (Figure 4F and G) or edelfosine (Figure 4H and I) showed enhanced sensitivity to high salt and drought stress. The seedling growth rate was significantly reduced by drought or salinity stress, and it was almost stagnated when the seedlings were further treated with the PI-PLC inhibitors, U73122 or edelfosine (*p*<0.01) (Figure 5A). The seedlings treated with both PI-PLC inhibitor and environment stress condition had lower fresh weight (FW) and relative water content (RWC) than those with environment stimuli only (*p*<0.01) (Figure 5B and C). In addition, biosynthesis of chlorophyll was more significantly inhibited and the malonaldehyde (MAD) contents were more accumulated in the both-treated-seedlings (*p*<0.01) (Figure 5D and E). Thus, the inhibition of TaPLC activity made the seedlings more sensitive to salt and drought stress.

### Expression pattern of *TaPLC1* under high salinity stress

**Figure 6:**
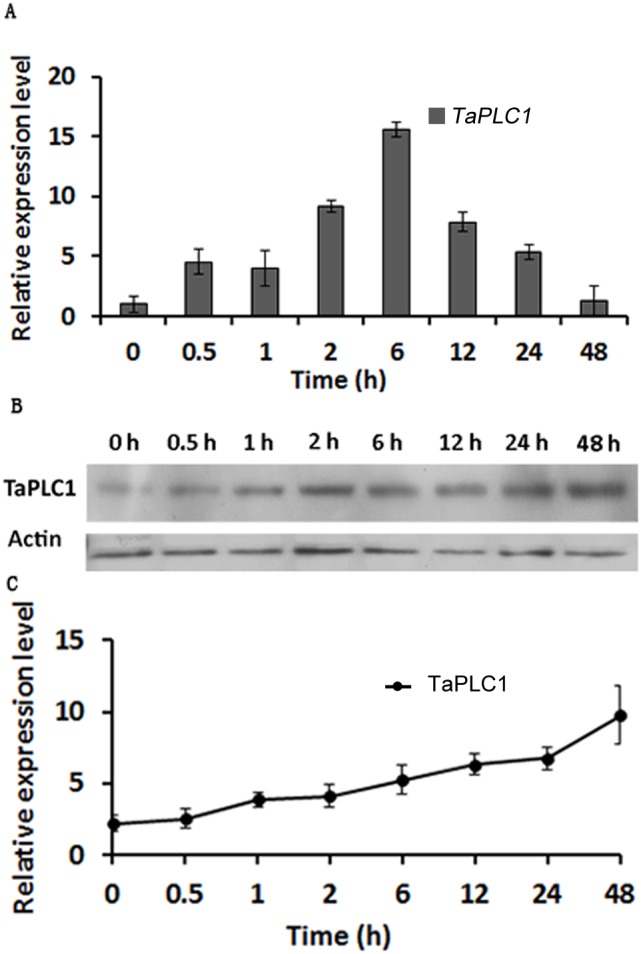
The *TaPLC1* gene expression pattern under salt stress. (A) The transcriptional expression pattern of *TaPLC1* under salt stress. First-strand cDNA was synthesized from 1 µg of total RNA and used to perform real-time RT-PCR with gene-specific primers and 18S RNA as an internal control. Plants were analyzed after 0, 0.5, 1, 2, 6, 12, 24 and 48 h. The levels of expression were shown as relative to 0 h, which were set to 1.0. (B and C) The TaPLC protein expression pattern under salt stress. (B) Western blot analysis of TaPLC1 expression under salt stress. Anti-TaPLC1 antibodies were prepared as shown in Text S1 and Figure S1. Anti-actin antibodies were used as an internal control. (C) Plot of the average and standard deviation among three repeats.

To investigate the role of *TaPLC* in stress responses to salinity in wheat, we measured the changes in *TaPLC1* expression induced by high salinity stress both at the transcriptional and post-transcriptional levels. Total RNA isolated from plants treated with 200 mM NaCl for various time periods as described in the “Materials and methods” was used to analyze the transcription of *TaPLC1* using real-time RT-PCR. The real-tme RT-PCR data were analyzed to calculate the relative transcript levels, and the expression level of the gene in untreated wheat seedlings was arbitrarily set to 1.0. The relative amounts of *TaPLC1* transcripts are as shown in Figure 6A. Our results indicate that *TaPLC1* was induced within 0.5 h after salt stress exposure, with an expression level that was 5-fold higher than that in the control. *TaPLC1* expression reached its maximum level, which was 16-fold higher than that in the control, at 6 h and then began to decrease (Figure 6A). We further examined the expression of the protein using Western blotting. Antibodies against TaPLC1 were prepared against a specific epitope of TaPLC1 (Text S1B). Our results show that the TaPLC protein level increased steadily from 0.5 to 48 h after salt treatment (Figure 6B and C). These results confirm that high salinity stress induced a rapid and lasting increase in *TaPLC1* expression.

### Expression pattern of *TaPLC1* under drought stress

**Figure 7:**
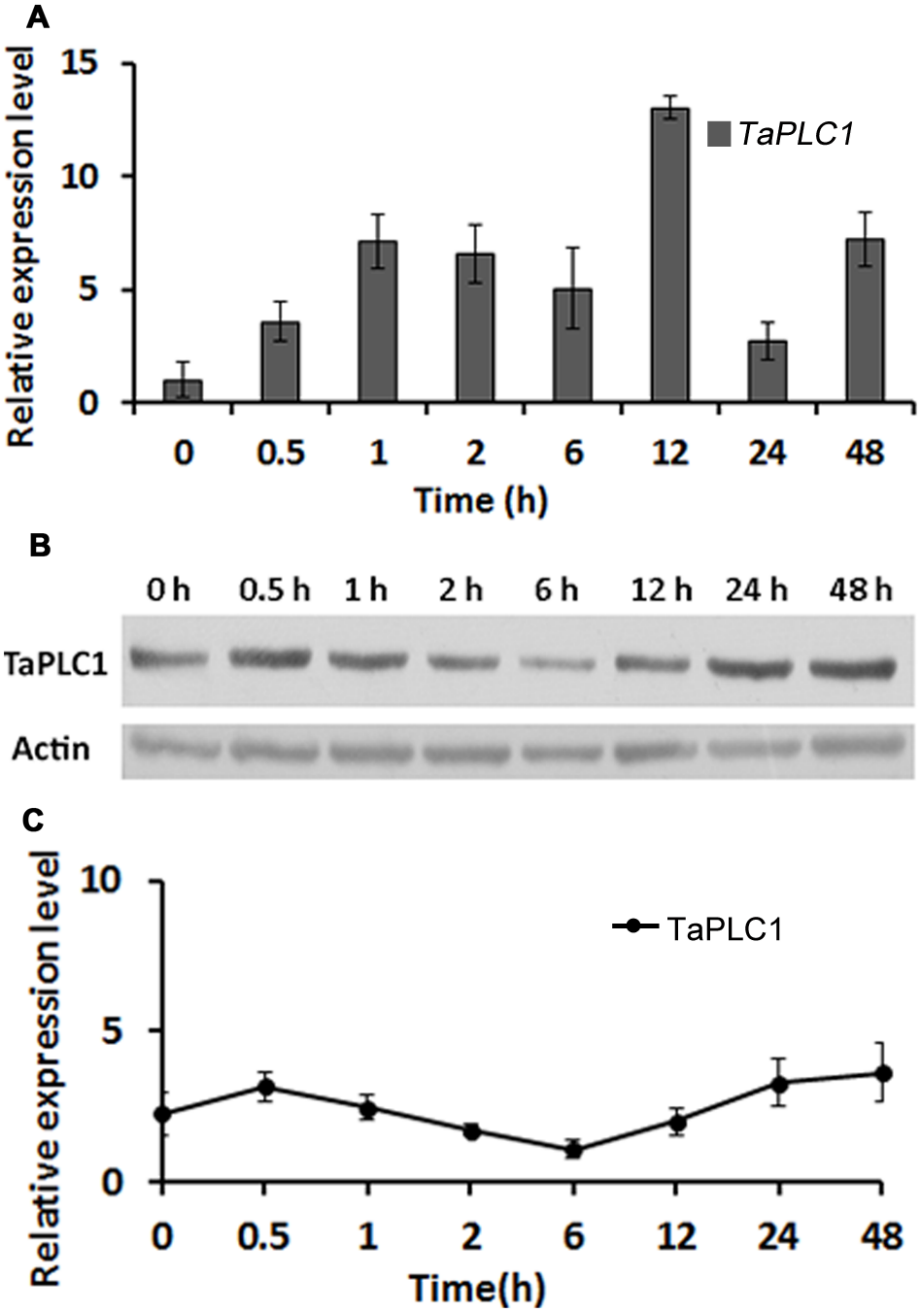
The *TaPLC1* gene expression pattern under drought stress. (A) The transcriptional expression pattern of *TaPLC1* under drought stress. First-strand cDNA was synthesized from 1 µg of total RNA and used to perform real-time RT-PCR with gene-specific primers and 18S RNA as an internal control. Plants were analyzed after 0, 0.5, 1, 2, 6, 12, 24, and 48 h. The levels of expression were shown as relative to 0 h, which were set to 1.0. (B and C) The TaPLC protein expression pattern under drought stress. (B) Western blot analysis of TaPLC1 expression under drought stress. Anti-TaPLC1 antibodies were prepared as shown in Text S1 and Figure S1. Anti-actin antibodies were used as an internal control. (C) Plot of the average and standard deviation among three repeats.

The *TaPLC1* expression pattern under drought stress was examined using the strategy described above. We found that *TaPLC1* was induced within 0.5 h after treatment with 20% PEG 6000, and that the expression level was 3-fold higher than that in the control (Figure 7A). This result is different from that obtained following salt exposure; *TaPLC1* expression decreased slightly at 6 h and reached its maximum level, which was 16-fold higher than that in the control, at 12 h (Figure 7A). In accordance with this, the TaPLC1 protein concentration showed largely the same changes by Western blotting (Figure 7B and C). These results indicate that *TaPLC1* is also involved in the response of wheat to drought stress, but that the expression pattern of *TaPLC1* in response to drought is different from that in response to salt stress.

## Discussion

### *TaPLC* is involved in wheat growth and the response to salt and drought stress

Plants respond to environmental stresses at both the cellular and molecular levels by altering the expression of many genes via complex signaling pathways. The role of *PI-PLC* genes in plant development is multifaceted. Over-expression of *BnPLC2* was found to cause an early shift from vegetative to reproductive growth, with shorter maturation periods [40]. StPLCs participate in cell cycle progression through DNA synthesis control in tobacco [41], while ZmPLC1 influences asymmetric cell division leading to the production of stomatal complexes in *Zea mays* [42]. Plant PLCs have been well studied during polarized pollen growth [17]. In the present study, we found that TaPLC is involved in regulating wheat seedling growth and the response to salt and drought stress using U73122 and edelfosine, both are specific antagonist of PI-PLC. First, U73122 or edelfosine inhibited seedling growth, the shoot length and fresh weight n°p seedling of treated-seedlings were both lower than control, and slow-growing seedlings had lower RWC (Figure 3). Second, seedlings treated with U73122 or edelfosine showed enhanced sensitivity to salt and drought stress. After 6 h of exposure to 200 mM NaCl or 20% PEG 6000, wilting was observed at the leaf tips in wheat seedlings (Figure 4). Six days later, all of the seedlings showed evident damage, with a growth decrease and other physiological phenotypes, such as wilting leaves caused by lower RWC, yellow leaves caused by lower content of chlorophyll and higher MAD contents indicate the damage of membrane (Figure 4 and Figure 5). However, the seedlings were still alive, and the inhibitory effect of stress on seedling growth was ameliorated when the stress was removed. In comparison, those seedlings whose leaves were treated with U73122 or edelfosine showed a more significant decrease of seedling length and fresh weight, and greater changes of the physiological parameters, such as much lower RWC or chlorophyll and higher content of MDA than before; finally the leaves turned brown, completely collapsed and died under the same conditions (Figure 4 and Figure 5). These results confirm the regulatory effect of PI-PLC on plant growth in response to salt and drought stress.

Pharmacological approaches for detection of gene function have been developed for a long time. There are two inhibitors for PI-PLC, U73122 and edelfosine, and both of them are often used to test the role of PI-PLC both in animals and plants [39]. Wong et al. [43] compared the effects of U73122 and the edelfosine on cytokinesis in cranefly and Drosophila spermatocytes. Their data showed that the effects of U73122 are indeed via PLC because U73122 and edelfosine produced similar effects on cell morphology and actin cytoskeleton organization. Djafi et al. [39] detected and evaluated the two inhibitors in identify genes regulated by a basal PI-PLC activity and showed that the effects of edelfosine and of U73122 are not independent, but inhibition of PI-PLC are common action. Till recently, U73122 was considered and used as the archetypal PI-PLC activity inhibitor and have been more used to research the plant PLC function [39], especially of participated response to abiotic stress, such as osmotic stress [35], [44], [45], heat stress [46] and cold stress [47]. Here we provided the evidence using U73122 and edelfosine that TaPLCs involved in the growth of wheat seedlings and response to salt and drought stress. It is noted that neither U73122 nor edelfosine has effect on seed germination (Figure 3 and Figure S2). However, the pharmacology experiments couldn’t confirm the PLC has no effect on seed germination. Because the inhibitors might not penetrate the seed coat and inhibit the role of PLC.

### TaPLC1 expression is induced by high salinity and drought stress

Many studies of plant stress have been aimed at identifying, cloning, and characterizing new genes involved in the response to salt and drought [48]. Plants perceive stress signals and respond through several complex adaptation mechanisms; one of the most important is signal reception and transduction, which produces significant changes in the expression of signaling molecules [48]. Several isoforms of plant PLCs have been cloned from a number of species, and PLC signal systems have been shown to be involved in responses to various environmental stresses [8], [9], [30], [49]. The function of plant PLCs in response to stress is usually assessed using transcriptome data. The *Arabidopsis* genome contains nine *AtPLC* genes. *AtPLC4*, *AtPLC5*, and *AtPLC7* show similar stress-related induction patterns, including strong induction by salt and drought [13], [37], [49]. *ZmPLC1* from maize (*Zea mays L*.) is up-regulated in response to dehydration [50], and enhanced expression of sense ZmPLC1 has been shown to improve the drought tolerance of maize plants [19], [51]. In this study, we found that *TaPLC1* expression in wheat was induced in response to high salt and drought stress at both the mRNA and protein levels. *TaPLC1* expression was very low in wheat seedlings under normal growth conditions (Figure 2), but was rapidly up-regulated under conditions of stress. The 16-fold change in *TaPLC1* expression was determined relative to untreated control plants and exhibited similarities with the results obtained for old leaves (Figure 6 and 7). These results imply that a rise in the expression of TaPLC1 in seedlings is an adaptive mechanism of plant development and the response to environmental changes. In plants, the PI-PLC isoform with the lowest expression level appears to be most susceptible to environmental stress. For example, all three *StPLC* genes are expressed in various tissues of potato; however, *StPLC2* mRNA expression was the lowest in expanded leaves of potato plants and was strongly induced by both wounding and wilting [13]. *Vigna radiata L.* also contains three *VrPLC* genes. Of these, *VrPLC3*, which exhibits the lowest level of transcription under normal growth conditions, is rapidly induced under conditions of drought and high salinity stress [15].

The various stress-responsive genes identified to date can be broadly categorized as early and late induced genes. The early genes, which encode many signaling components, are induced within minutes of stress signal perception and are often expressed transiently. Late induced genes are activated by stress more slowly, usually after hours of stress perception, and the expression of these genes is often sustained [52]. *TaPLC1*, which encodes a signaling protein, seems to be both an early and late induced gene. The expression level of *TaPLC1* was rapidly induced several fold under salt and drought stress within 30 min, and 16-fold after 6 h (salt stress) or 12 h (drought stress). This is perhaps due to the various functions of the phosphoinositide signal system. *Myo*-inositol (Ins) derivatives represent a large family of molecules, many of which are involved in the response to osmotic stress. Some, such as IP6, the product of the stepwise phosphorylation of IP3, have two separate functions: signaling and storage. Storage IP6 is a source of both phosphate and Ins in storage tissues and can be used to produce compatible solutes as osmoprotectant to increase a plant’s salt or drought tolerance. InsP6 signalling functions are short-ranged and much faster, such as to release intracellular Ca^2+^, to affect local gene expression and to activate F-box related signalling pathways [6].

In addition, evidence suggests that the phosphoinositide signaling system in wheat is involved in the response of plants to salt and drought. Recently, a proteomic study of a tolerant genotype of durum wheat under salt-stress conditions showed significant changes in the expression of 83 proteins at high levels of salinity. One of these, inositol 3-phosphate synthase, is an enzyme that plays a critical role in Ins biosynthesis; it was down-regulated at high levels of salinity [48]. Phosphatidylinositol (PI) 4 kinases (PI4Ks) generate PI4-phosphate (PI4P), the precursor of regulatory phosphoinositides. PI4P is one of the substrates of PLC [53]. A stress-inducible type II PI4K gene named TaPI4KIIγ was obtained by the *de* *novo* transcriptome sequencing of drought-treated wheat (*T. aestivum*). Over-expression of TaPI4KIIγ revealed that the protein enhanced drought and salt stress tolerance during seed germination and seedling growth [36]. DAG, another product of PI-PLC is further phosphorylated to PA via DAG kinase (DGK). Accumulating evidence suggests that PA plays a pivotal role in the plant’s response to environmental signals [54]. Recently, Djafi et al. [39] provided with suspension cells and seedlings of *Arabidopsis* that the inhibitors of PI-PLCs could also inhibit PA formation, showing that basal PI-PLCs act on gene expression through their coupling to DGKs, and some DREB2 genes, encode transcription factors with major roles in responses to environmental stresses, are up-regulated in presence of the inhibitors. Our expression data for *TaPLC1* confirm its role in the regulation of seedling growth and the response to salt and drought stress in *T. turgidum*. Although numerous studies have been conducted to understand how plants deal with salt and drought stress, many questions remain, including the mechanism by which these lipids signal and how osmotic stress activates phosphoinositide signaling [6]. These questions will form the basis for future research and the components of phospholipids signals, such PA and DREB2, would be our concerns for further research. In addition, the activity of TaPLC will also be one of the priorities of our future work.

## Supporting Information

Figure S1 **The production of antibodies against TaPLC1.** (A) Agarose gel electrophoresis was performed to identify the pMD19-T-*TaPLC1* plasmid digested with *Eco*RI and *Xho*I. The fragment was detected as a band of the expected size (600 bp). The molecular mass is indicated on the left. (B) Identification of TaPLC1 (amino acids 151–350), which was cloned into pET-30a, by 12% SDS-PAGE. Compared with the control (1; without IPTG treatment), the sample (2; with IPTG treatment) could be detected as a 40-kDa band. The molecular mass is indicated in the middle (in kDa). (C) SDS-PAGE was performed to identify the TaPLC1 fragment (amino acids 151–350) purified from bacteria. An obvious band of the expected size (40 kDa) was purified from the precipitate (2), compared with the control (1), which was purified from the supernatant liquor. The molecular mass is indicated on the left in kDa.
(TIF)

Figure S2 **The role of U73122 or edelfosine on seeds germination.** U73122 or edelfosine treated the seeds for 12 h (A), 24 h (B) and 36 h (C). U73122 or edelfosine has no effect on seeds germination compared with the control.
(TIF)

Table S1 **The anti-PLC1 antibody titer.** Water and preimmune serum were used in the titer test as an assay control and negative control, respectively.
(DOCX)

Text S1 **The fragment sequences amplified from wheat genomic DNA.** The 600-bp fragment amplified from wheat genomic DNA using the primers Anti-PLC1 F and Anti-PLC1 R (A) and corresponding to amino acids 151–350 of TaPLC1 (B).
(DOCX)
