# Supplementary figures and images for: Expression Analysis of a Stress-Related Phosphoinositide-Specific Phospholipase C Gene in Wheat (Triticum aestivum L.)
Source: PLoS One. 2014 Aug 14;9(8):e105061. doi: 10.1371/journal.pone.0105061 (PMC4133336; doi:10.1371/journal.pone.0105061)

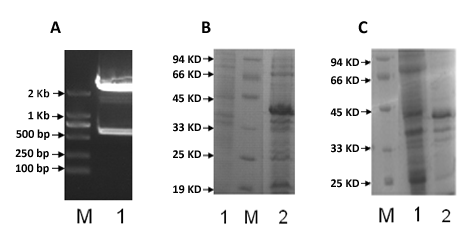

Supplement: Figure S1 — The production of antibodies against TaPLC1. (A) Agarose gel electrophoresis was performed to identify the pMD19-T-TaPLC1 plasmid digested with EcoRI and XhoI. The fragment was detected as a band of the expected size (600 bp). The molecular mass is indicated on the left. (B) Identification of TaPLC1 (amino acids 151–350), which was cloned into pET-30a, by 12% SDS-PAGE. Compared with the control (1; without IPTG treatment), the sample (2; with IPTG treatment) could be detected as a 40-kDa band. The molecular mass is indicated in the middle (in kDa). (C) SDS-PAGE was performed to identify the TaPLC1 fragment (amino acids 151–350) purified from bacteria. An obvious band of the expected size (40 kDa) was purified from the precipitate (2), compared with the control (1), which was purified from the supernatant liquor. The molecular mass is indicated on the left in kDa. (TIF) [file pone.0105061.s001.tif]

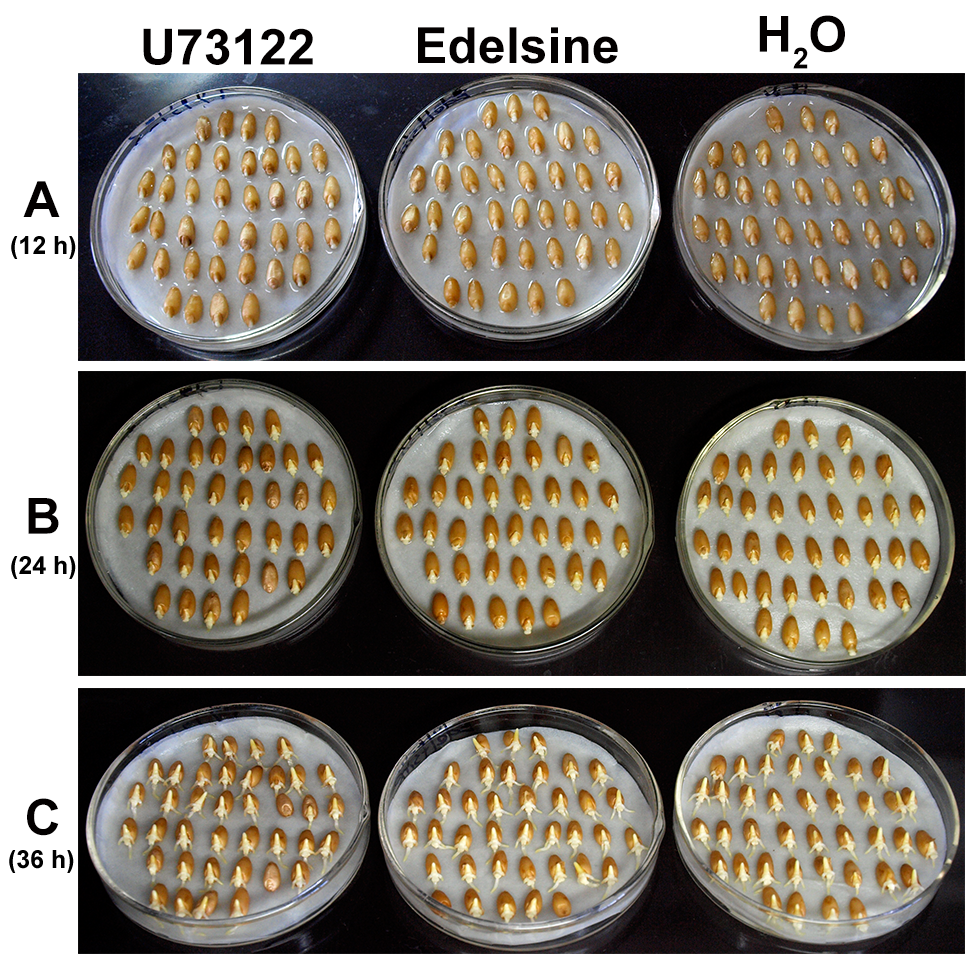

Supplement: Figure S2 — The role of U73122 or edelfosine on seeds germination. U73122 or edelfosine treated the seeds for 12 h (A), 24 h (B) and 36 h (C). U73122 or edelfosine has no effect on seeds germination compared with the control. (TIF) [file pone.0105061.s002.tif]
