# Supplementary material for: Expression Analysis of a Stress-Related Phosphoinositide-Specific Phospholipase C Gene in Wheat (Triticum aestivum L.)
Source: PLoS One. 2014 Aug 14;9(8):e105061. doi: 10.1371/journal.pone.0105061 (PMC4133336; doi:10.1371/journal.pone.0105061)
Supplement: Table S1 — The anti-PLC1 antibody titer. Water and preimmune serum were used in the titer test as an assay control and negative control, respectively. (DOCX) [file pone.0105061.s003.docx]

**Table S1**. The anti-PLC1 antibody titer.

| Dilution ratio(×10^2^) | | 2 | 4 | 8 | 16 | 32 | 64 | 512 | Assay control | Negative control |
| --- | --- | --- | --- | --- | --- | --- | --- | --- | --- | --- |
| Titer | 1.300 | | 1.275 | 1.263 | 1.086 | 1.104 | 1.044 | 0.804 | 0.017 | 0.102 |
