# Supplementary material for: Expression Analysis of a Stress-Related Phosphoinositide-Specific Phospholipase C Gene in Wheat (Triticum aestivum L.)
Source: PLoS One. 2014 Aug 14;9(8):e105061. doi: 10.1371/journal.pone.0105061 (PMC4133336; doi:10.1371/journal.pone.0105061)
Supplement: Text S1 — The fragment sequences amplified from wheat genomic DNA. The 600-bp fragment amplified from wheat genomic DNA using the primers Anti-PLC1 F and Anti-PLC1 R (A) and corresponding to amino acids 151–350 of TaPLC1 (B). (DOCX) [file pone.0105061.s004.docx]

**Text A**. The fragment amplified from wheat genomic DNA.

GCCAAGGATGGTGGTGCCGCGAAAGACGGTGATGCGGAGCAGAATCCTGGCAAAGGAACTGACGATGATGCGGCTTGGGGAACAGAAGTCCCAGATTTCAAGACTGAAATCCAATCTGCTAAAGAAGATGATGCCTCAGAGAACCGTAGAGACGGCGATGAGGACGACGACGATGAGGACGAACAGAAAATGCAACAGCATCTAGCTCCACAGTATAAGCGCCTTATTACTATAAGAGCAGGAAAGCCAAAGGGGGGTACTACGTCTGATGCCTTGAAGTGTGACCCGAACAAAGTTAGGCGGCTCAGTTTGAGCGAGCAACAGCTTGCCAAAGCTGTAGTTAATCATGGCACCGAAATAGTGAGGTTTACACAGAGGAATCTACTGAGGATATACCCAAAGGGCACTCGGGTTACTTCATCCAACTACAATCCATTTATTGGTTGGGTGCATGGTGCTCAGATGGTAGCCTTCAATATGCAGGGATATGGAAGAGCTCTTTGGTTAATGCATGGATTTTATAAAGCCAATGGTGGCTGTGGCTACGTGAAGAAACCGGATTTCTTGATGCAGTCCGAGCCGGAAGTTTTCGATCCAAAA

**Text B**. The amino acids 151‒350 of TaPLC1.

AKDGGAAKDGDAEQNPGKGTDDDAAWGTEVPDFKTEIQSAKEDDASENRRDGDEDDDDEDEQKMQQHLAPQYKRLITIRAGKPKGGTTSDALKCDPNKVRRLSLSEQQLAKAVVNHGTEIVRFTQRNLLRIYPKGTRVTSSNYNPFIGWVHGAQMVAFNMQGYGRALWLMHGFYKANGGCGYVKKPDFLMQSEPEVFDPK
